# Triangle Tilt Surgery: Effect on Coracohumeral Distance and External Rotation of the Glenohumeral Joint

**Published:** 2010-11-19

**Authors:** Rahul K. Nath, Faiz Mahmooduddin

**Affiliations:** Texas Nerve and Paralysis Institute, 6400 Fannin St, Ste 2420, Houston, TX

## Abstract

**Objective:** Shoulder muscle imbalances and bone deformities that develop secondary to obstetric brachial plexus injury have been extensively studied. Less emphasis has focused on coracohumeral distance, a small value potentially being linked to impaired shoulder external rotation. The purpose of this study is to analyze coracohumeral distances and shoulder external rotation in obstetric brachial plexus injury patients before and after triangle tilt surgery. **Methods:** Twenty patients with deformities secondary to obstetric brachial plexus injury were included. Coracohumeral distances were measured on computed tomographic images. Clinical functioning was evaluated through video recordings by using a modified Mallet scale. Paired Student *t* tests were used to determine statistical significance of anatomic and functional parameters, pre- and postoperatively. **Results:** Coracohumeral distance (*P* < .0006), total Mallet score (*P* < .0001), supination angle (*P* < .0001), and individual Mallet scores for all external rotation parameters including hand-to-mouth (*P* < .0001), supination (*P* = .0010), external rotation (*P* < .0001), hand-to-neck (*P* < .0001), and hand-to-spine (*P* = .0064) were significantly higher postoperatively than preoperatively for affected shoulders. Hand-to-mouth angles were significantly lower postoperatively than preoperatively (*P* < .0001). Coracohumeral distance in unaffected shoulders remained unchanged. **Conclusions:** Triangle tilt surgery significantly improves coracohumeral distance and clinical functioning in obstetric brachial plexus injury patients. Coracohumeral distance plays a key role in shoulder external rotation. Increasing coracohumeral distance significantly improves all external rotation parameters and total Mallet scores. The triangle tilt surgery relieves excessive tightness of the anterior stabilizing complex, widens coracohumeral distance, and improves external rotation of shoulder.

Obstetric brachial plexus injuries (OBPI) can involve injury to any or all of the C5-T1 nerve roots, but most commonly includes C5 and C6. Many patients recover spontaneously within the first 3 months of life. The major cause of long-term morbidity in this patient population can be attributed to secondary muscle imbalances and bone deformities at the shoulder joint due to incomplete and asymmetric recovery of neurological function.^1^^-^3 Dislocation and posterior subluxation of the humeral head, glenoid retroversion, and glenohumeral dysplasia are characteristic anatomical changes of the shoulder joint. Considerable efforts have been made to characterize and correct the glenohumeral deformities in OBPI patients. However, relatively less emphasis has been placed on other important factors such as the distance between the coracoid process and the humeral head, or coracohumeral distance (CHD). We hypothesize that CHD plays an important role in external rotation and that the triangle tilt (TT) surgery increases CHD, and effectively, external rotation parameters as well. Abnormally short values of CHD may be due to deformities involving the coracoid process among other reasons.^4^ We believe that a major contributing factor leading to a short CHD is a shortening of the anterior stabilizing complex (ASC) of the shoulder joint. We further believe that this shortened ASC impairs full external rotation movements of the affected shoulder joint. We propose that by lengthening the ASC, the tightening of the anterior glenohumeral ligament will be relieved, effectively widening the coracohumeral distance and improving external rotation functioning of the shoulder joint.

Ossification of the coracoid process begins with the development of a primary center at 3 to 4 months. By the age of 1 year, the primary ossification center of the coracoid process has enlarged, and the tip of the coracoid process is clearly identifiable radiographically. A bipolar physis is present between the primary coracoid center and the primary scapular center until late adolescence.^5^

Abnormal growth of the coracoid process has been observed in OBPI patients.^1^,^6^^-^10 In a study conducted by Birch,^1^ it was found that out of 166 patients with secondary bone deformities, 90 had moderate coracoid overgrowth and 36 had severe coracoid overgrowth. A classification system was developed by Kambhampati et al^8^ to grade coracoid deformities. Observing 183 patients intraoperatively, 57% of the patients were found to have grade 1 and 31% had grade 2 coracoid deformity (grade 0: normal coracoid; grade 1: moderately deformed coracoid with the tip at the level of capital physis; and grade 2: severely deformed coracoid with the tip below the level of capital physis). Despite these obvious abnormalities observed in the coracoid process, there are few studies that examine the implications of a shortened coracohumeral distance and the effect on clinical function.

The purpose of the this study is to analyze coracohumeral distances and shoulder external rotation function in obstetric brachial plexus injury patients before and after the TT surgery, which is composed of distal acromioclavicular triangle repositioning^11^ and shoulder ASC expansion and reconstruction. The effect of these interventions is to allow anterior migration of the humeral head, enhancing the chances of favorable glenohumeral remodeling.^12^

## METHODS

Twenty patients (10 men and 10 women) with coracohumeral shortening secondary to OBPI were included in this study. The patients had varied degrees of initial nerve injury, which involved the C5-C6, C5-C7, C5-C8, or C5-T1 roots. There were 13 right-sided and seven left-sided injuries. These patients underwent TT from January 2007 to March 2009. Their ages ranged from 15 months to 13 years at the time of the procedure, with the average age being 5 years.

**Figure 1:**
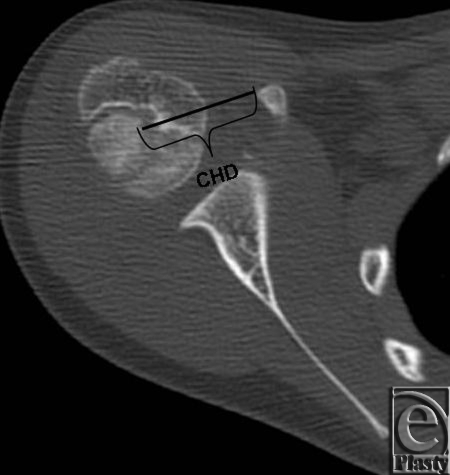
Representative axial computed tomographic image of the affected shoulder from a patient with obstetric brachial plexus injury. CHD represents the coracohumeral distance, measured from the coracoid process to the center of the humeral head.

In the current study, data were collected on both pre- and postoperative axial computed tomographic (CT) images of affected and unaffected shoulders. The average follow-up time from TT to postoperative CT was 18 months. Each axial CT image that was chosen clearly showed the center of the humeral head, the glenoid articulation with the humeral head, and the tip of the coracoid process. Coracohumeral distance was measured pre- and postoperatively to determine whether the TT procedure had an effect on the entity. The coracohumeral distance measurement technique was adapted from Gerber et al^13^ due to the incomplete ossification of the humeral head and the hypoplasia from the brachial plexus injury. It was defined as the distance from the tip of the coracoid process to the calculated center of the humeral head that was visible in CT images, instead of the subchondral bone of the humeral head (Fig 1).

**Figure 2:**
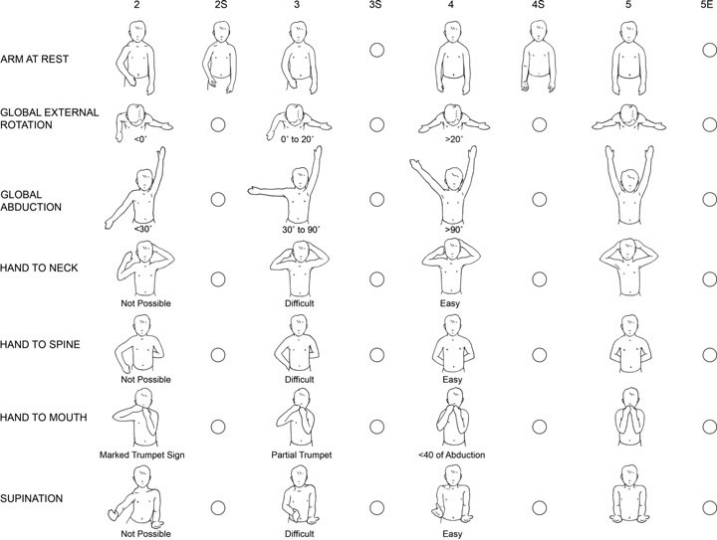
The Nath Modification of Mallet's System: clinical scoring of function. In addition to assessing the classical functions of the Modified Mallet system, supination and the resting position are evaluated. To further define deformity, fixed forearm supination (positions 2S, 3S, and 4S) as well as external rotation position (5E) are scored.

All the measurements were performed by a trained scientist (F.M.) independent of the surgeon and senior author. Graphic software (Universal Desktop Ruler, AVP-Soft.com, Voronezh, Russia) was used for all measurements made on axial CT images. Distance was measured in pixels and converted into standard length units as millimeters. Clinical functioning of patients was evaluated preoperatively and postoperatively through video recordings using a modified Mallet scale (Fig 2). Global abduction was not measured, as it is addressed with the modified quad surgery. The TT surgery is designed to address only external rotation deficiencies of shoulder.

The mean was calculated for each measured parameter. Paired student *t* tests were conducted to determine any significant differences between pre- and postoperative values for coracohumeral distance, total Mallet score, as well as all individual components of the Mallet score. Results were considered statistically significant at *P* < .05 level. All statistical analyses were performed using the Analyse-It plugin for Microsoft Excel 2003 software (Leeds, UK).

## RESULTS

Patients included in our study had varying degrees of secondary deformities and typically presented with fixed medial rotation contracture and scapular deformity with shortened coracohumeral distances of the affected shoulders as visible in CT images.

## Summary of Results for Measurement Parameters

The pre- and postoperative means, difference in means, and *P* values for each measurement parameter are shown in Table 1. Significant differences were found for affected shoulders between pre- and postoperative values of coracohumeral distance, total Mallet scores, and all external rotation parameters of the modified Mallet scale.

**Figure 3:**
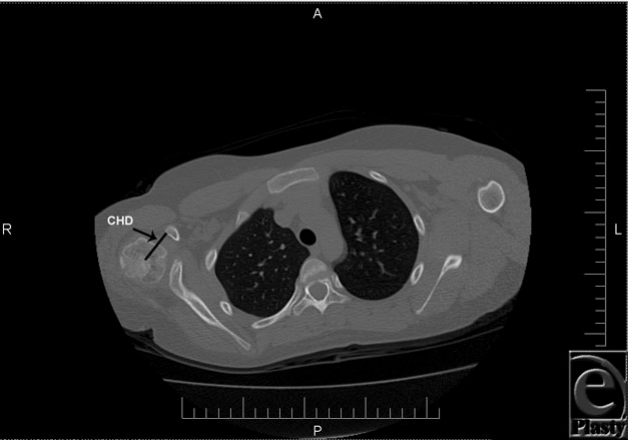
Preoperative computed tomography of a patient with obstetric brachial plexus injuries included in this study showing coracohumeral distance, labeled “CHD.” Scale is shown at bottom. Space between each hache mark is 10 mm. CHD = 35 mm.

**Figure 4:**
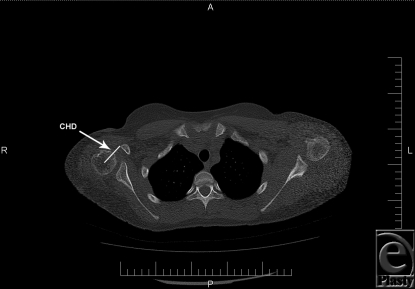
Postoperative computed tomography of the same patient showing coracohumeral distance, labeled “CHD.” Scale is shown at bottom. Space between each hache mark is 10 mm. CHD = 40 mm.

Coracohumeral distance, measured from the most proximal aspect of the coracoid tip to the calculated center of the ossified humeral head visible on the axial CT image, was significantly higher postoperatively than preoperative values (mean: 31.1 mm vs 27.6 mm; difference in means: 3.5 mm; *P* < .0006) for affected shoulders. Figures 3 and 4 show the pre- and postoperative CTs, respectively, with coracohumeral distances on the same OBPI patient. In the unaffected shoulders, coracohumeral distance remained unchanged during the same time frame.

Hand-to-mouth angles were significantly lower postoperatively that preoperatively (mean: 38°. vs 92°.; difference in means: 54°.; *P* < .0001). Correspondingly, the postoperative hand-to-mouth Mallet scores were significantly higher than the preoperative values (mean: 3.7 vs 2.1; difference in means: 1.6; *P* < .0001).

Postoperative supination angles were significantly higher than their respective preoperative values (mean: 52°. vs −2°.; difference in means: 54°.; *P* < .0001). The corresponding Mallet scores for supination were significantly higher postoperatively than preoperatively (mean: 3.9 vs 2.8; difference in means: 1.1; *P* = .0010).

Postoperative external rotation, hand-to-neck, and hand-to-spine scores were significantly higher than their respective preoperative values. Post–total Mallet scores were significantly higher than pre–total Mallet scores (mean: 18 vs 12; difference in means: 6; *P* < .0001).

## Discussion

We measured coracohumeral distances on CT images, scored external rotation clinical functioning parameters, and compared results obtained preoperatively with those obtained postoperatively. Significant differences for all measurement parameters were found between the pre- and postoperative values of affected shoulders. Values of coracohumeral distance, supination angles, and Mallet scores (hand-to-mouth, supination, external rotation, hand-to-neck, hand-to-spine, and total) were significantly higher postoperatively than preoperatively, whereas values of hand-to-mouth angles were significantly lower postoperatively than preoperatively. All of these measurements are consistent with impingement in function of the injured extremity.

The results show that the coracohumeral distance significantly increased in the affected shoulders postoperatively. Coracohumeral distance remained unchanged in the unaffected shoulders during the same time frame. This leads to the conclusion that the increased CHD on the operated side is due to the TT surgery because that was the only intervention. Obstetric brachial plexus injuries affect not only the glenohumeral joint but also the spatial relationship between the coracoid process and the humeral head. The coracohumeral distance is also referred to as the subcoracoid space in another study.^13^ Our study demonstrates the importance of subcoracoid space widening and its positive effects on clinical functioning in OBPI patients.

The TT surgery significantly improves coracohumeral distance and clinical functioning in OBPI patients. The distance between the coracoid process and the humeral head plays a key role in the clinical functioning of OBPI patients, particularly in external rotation movements of the shoulder. By increasing the coracohumeral distance, significant improvements in individual external rotation parameters and total Mallet scores were noted. The TT surgery allows for the anterior normalization of the posteriorly subluxed humeral head^12^ in 2 ways: (1) by anatomically repositioning the abnormal forward tilt of the acromioclavicular triangle and (2) by releasing the shoulder's ASC tightness, which passively restricts external rotation of the humeral head. This ASC tightness causes the coracoid process to angulate toward the humeral head (“beaking” of the coracoid process).

The ASC tightness is caused by contracture of the subscapularis, pectoralis minor, and short head of the biceps muscle/tendon units (the dynamic component) and by passive shortening of the anterior ligaments and capsule of the shoulder joint (the static component) in response to the elevation and lateral rotation of the scapula (the SHEAR deformity: Scapular hypoplasia, elevation and rotation).^14^ The TT surgery relieves the excessive tightness of ASC dynamic and static components, effectively widening the coracohumeral distance, allowing anterior migration of the humeral head^12^ and improving functional external rotation of the shoulder. Ultimately, this allows for congruency of the glenohumeral joint, as well as favorable anatomic remodeling of the glenoid fossa.

The coracohumeral shortening may be attributed to the tension induced by the medial rotation contracture and/or the posterior subluxation of the humeral head that is placed on the coracoid process.^8^,^15^ This is secondary to the presence of the SHEAR deformity. Therefore, increasing the coracohumeral distance not only allows for more space and an increased range of motion of the humeral head anteriorly, which increases external rotation of the shoulder, but also leads to eventual glenohumeral anatomical normalization.^16^ The longer the coracohumeral distance, the greater the outcomes are in terms of clinical functioning.

Shoulders exhibiting severe posterior subluxation of the humeral head usually present with smaller subcoracoid spaces. As abnormal development progresses, the coracoid process approaches the humeral head, not by actual impingement onto the humeral head itself, but rather into the subcoracoid space due to the frequent presence of posterior subluxation of the humeral head in the affected shoulders. The impingement of the coracoid process into the subcoracoid space becomes even more pronounced when the shoulder is internally rotated, as evidenced in the study conducted by Gerber et al.^4^

Surgical intervention is necessary for these patients to correct their glenohumeral deformity by repositioning the posteriorly subluxed humeral head anteriorly to articulate properly with the glenoid.^12^ There is no medical literature showing that nonsurgical treatment has any benefit or indeed, any influence, on the development of the glenohumeral articulation in these children. The abnormal position of the coracoid process is important in the success of the TT surgery. Therefore, it is essential to understand the spatial relationship of the coracoid process to the humeral head prior to surgical treatments. It is important to know that the worse the glenohumeral deformity is, the smaller is the subcoracoid space, and the more severe is the impingement.^13^

We recommend the TT surgery to correct both the glenohumeral deformity and the abnormal spatial relationship of the coracoid process to the humeral head. The TT surgery achieves these results through combining bony repositioning of the distal acromioclavicular triangle with ASC lengthening. The TT surgery allows for the successful repositioning of the posteriorly subluxed humeral head anteriorly to articulate properly with the glenoid and to reverse the effects of glenohumeral deformity,^12^ which caused the coracoid process to protrude toward the humeral head. The ASC lengthening relieves the tightening of the anterior glenohumeral ligament, effectively widening the coracohumeral distance and allowing anterior migration of the humeral head within the glenoid fossa.^12^

## Figures and Tables

**Table 1:** Student t test to determine statistical significance of differences between pre- and postoperative values of anatomic and functional parameters*

Parameter	Preoperative Mean	Postoperative Mean	Difference in Means	*P*†
Coracohumeral distance, mm	27.6	31.1	3.5	.0006
External rotation score (1-5)	2.4	3.7	1.3	<.0001
Hand-to-mouth angle, degree	92	38	54	<.0001
Hand-to-mouth score (1-5)	2.1	3.7	1.6	<.0001
Hand-to-neck score (1-5)	2.5	3.7	1.2	<.0001
Hand-to-spine score (1-5)	2.3	2.8	0.5	.0064
Supination angle, degree	t2	52	54	<.0001
Supination score (1-5)	2.8	3.9	1.1	.0010
Total Mallet score	12	18	6	.0001

*Comparison of coracohumeral distance and modified Mallet score parameters before and after triangle tilt surgery in patients with obstetric brachial plexus injuries. Angle of apparent active supination was recorded as follows: 0°. = neutral position, 90°. = full apparent supination, and −90°. = full apparent pronation.

†Statistically significant difference in means (*P* < .05).
